# A Gastrointestinal Manifestation of Primary Cutaneous Aggressive Epidermotropic Cytotoxic T-Cell Lymphoma: A Case Report

**DOI:** 10.7759/cureus.71953

**Published:** 2024-10-20

**Authors:** Max E Edeson, Ethan Burg, Gabrielle K Sharbin, Davood K Hosseini, Jose Churrango

**Affiliations:** 1 Internal Medicine, Hackensack Meridian Health, Hackensack Meridian School of Medicine, Nutley, USA; 2 Internal Medicine, Hackensack Meridian Health, Hackensack University Medical Center, Hackensack, USA; 3 Gastroenterology, Hackensack Meridian Health, Palisades Medical Center, North Bergen, USA

**Keywords:** aggressive epidermotropic cytotoxic t cell lymphoma, cutaneous t cell lymphoma, duodenitis, gastric erosion, gastric ulcer, gastritis, t cell lymphoma

## Abstract

Cutaneous T-cell lymphomas (CTCL) are the most frequently encountered form of primary cutaneous lymphoma (PCL). Although this group of diseases primarily affects the skin, extracutaneous manifestations are reported. Primary cutaneous aggressive epidermotropic cytotoxic T-cell lymphoma (pcAECTCL) is a rare form of CTCL that usually presents with an aggressive course and poor prognosis. Given the scarcity of cases, the disease is poorly understood. There have been reports of cases of extracutaneous manifestations of the disease, with more aggressive courses associated with an increased propensity for systemic spread. The most common extracutaneous manifestations of pcAECTCL include those affecting the lungs, central nervous system, testis, and oral mucosa, which can cause progressive dysphagia. We describe a patient with a chronic relapsing/remitting rash diagnosed as pcAECTCL. A positron emission tomography (PET) scan revealed diffuse uptake within the gastric region. Initial esophagogastroduodenoscopy (EGD) showed multiple ulcerations and erosions in the stomach with concern for extracutaneous spread of the patient’s pcAECTCL. However, no definitive histopathological or flow immunophenotypic evidence of metastasis was found on the initial or subsequent repeat EGD.

## Introduction

Primary cutaneous lymphomas (PCLs) are a form of extranodal non-Hodgkin's lymphoma that serves as a unifying term for a rare group of diseases derived from clonal proliferation of B or T lymphocytes [1-3]. The monoclonal proliferation of T lymphocytes in cutaneous lymphomas is categorized as cutaneous T-cell lymphoma [1-3]. This accounts for 83% of PCLs [3]. CTCL itself encompasses a varying group of rare pathologies with the most frequently encountered being mycosis fungoides [1,3]. CTCL is a disease that primarily affects the skin but has the potential for extracutaneous manifestations [1-3]. Extracutaneous manifestations develop in 30% of cutaneous lymphomas as a result of a progression of primary skin lesions, and this extracutaneous spread can both involve or spare lymph nodes [3,4].

Primary cutaneous aggressive epidermotropic cytotoxic T-cell lymphoma (pcAECTCL) is an extremely rare form of CTCL, representing less than 1% of all CTCL [5]. It has a predominance in males and most frequently presents in elderly patients with a mean age of 77 [1,4-6]. Given the rarity of pcAECTCL, it is poorly characterized [6]. The prognosis of pcAECTCL is poor, as it typically follows an aggressive course with a high propensity for extranodal spread and unfavorable response to typical CTCL therapies [1,4-6]. There have been cases reported of aggressive cytotoxic CTCL developing from a previously indolent course of chronic patches [1,4-7]. However, most frequently, pcAECTCL presents as rapidly evolving, widely distributed papules, patches, nodules, tumors, and plaques that frequently ulcerate. Lesions may present more frequently at acral sites [1,4,7]. Oral mucosal involvement is also common and reflects a poorer prognosis [4-6]. These oral lesions have been reported to cause progressive dysphagia [4-6]. The most common sites involved in pcAECTCL include the lungs, central nervous system, and testis, with the lymph nodes often being spared [4].

Moreover, aggressive cases presenting with widespread ulcerative cutaneous lesions have an increased likelihood of systemic spread [4]. Although not a common site for metastasis, as potentially seen in this case, the gastrointestinal tract may represent a rare extracutaneous manifestation of pcAECTCL.

## Case presentation

**Figure 1:**
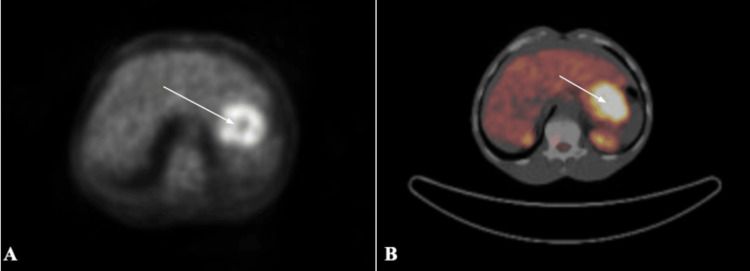
Initial PET/CT (a) CT scan axial view at the level of the abdominal viscera showing increased uptake in the gastric region, (b) F18 FDG PET fused axial imaging showing increased diffuse appearing uptake throughout the gastrum with a max SUV of 7.2. Given this increased uptake in the setting of the patient’s abdominal pain and active malignancy, there was a concern for this being representative of an extracutaneous manifestation of his pcAECTCL in his GI tract. PET/CT: positron emission tomography/computed tomography; F18 FDG PET: fludeoxyglucose-F18 positron emission tomography; SUV: standardized uptake value; pcAECTCL: primary cutaneous aggressive epidermotropic cytotoxic T-cell lymphoma

A 58-year-old male, with a history of chronic relapsing rash, presented to the Emergency Department (ED) with a one-month history of dysphagia, odynophagia, epigastric tenderness, and 30 lbs weight loss. The patient endorsed fatigue but denied fevers, night sweats, nausea/vomiting, blood in the stool, or changes in bowel habits. The patient also denied recent nonsteroidal anti-inflammatory drug (NSAID) usage or personal or family history of cancer. Vital signs were stable, showing BP 122/71 mmHg, pulse 60 bpm, respiration 18 rpm, temperature 98.3 °F (36.8 °C), and oxygen saturation (SpO2) 100%. An examination showed epigastric tenderness, left costo-vertebral tenderness, and diffuse maculopapular rash with ulcerations on the back, trunk, upper and lower extremities. Remarkable findings included hypoproteinemia of 5.3 g/dL and hypoalbuminemia of 2.3 g/dL. Given the patient's epigastric abdominal pain with significant weight loss and nausea, CT abdomen/pelvis (CT A/P) with contrast was performed due to concern for underlying malignancy. CT A/P showed no definite findings of malignancy in the abdomen or pelvis. Equivocal CT results prompted a PET scan, which showed abnormal uptake in the gastric region that had changed from earlier (Figure 1).

**Figure 2:**
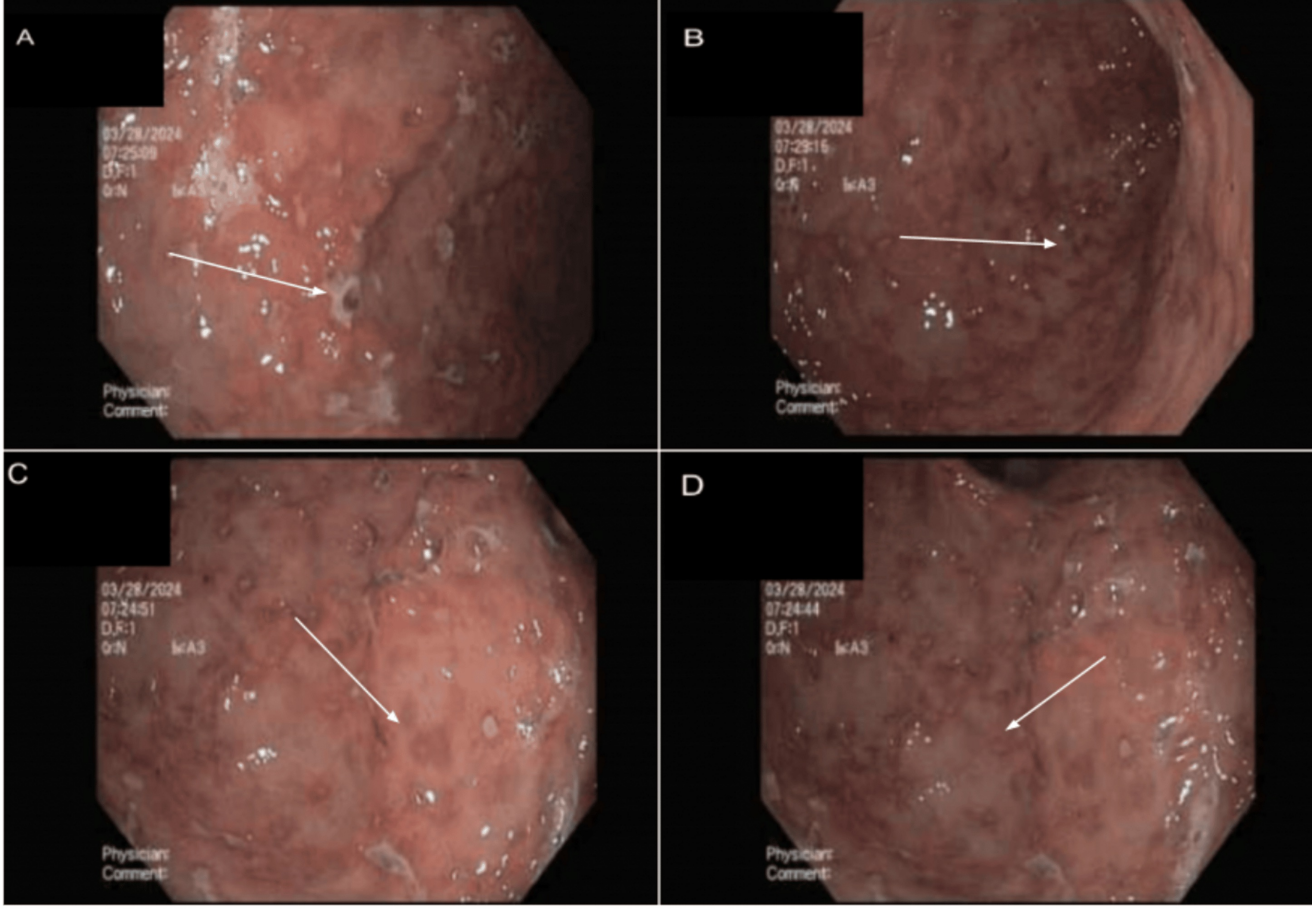
Initial EGD with biopsy (a-b) Multiple ulcerations and erosions are seen in the gastric body, (c-d) Multiple ulcerations and erosions are also shown in the fundus. Ulcerations and erosions are denoted by white arrows indicating disruptions in the normal pink-appearing gastric mucosa instead of showing red, white, or dark/black lesions with an erythematous rim. Biopsies of these lesions were taken with cold forceps; subsequent histology was negative for lymphoproliferative disorder. EGD: esophagogastroduodenoscopy

**Figure 3:**
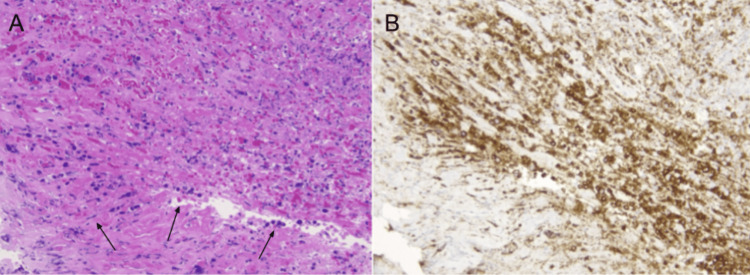
Histopathology of the gastric biopsy (a) Gastric biopsy with Hematoxylin & Eosin (H&E) staining, obtained from the initial esophagogastroduodenoscopy (EGD). Arrows highlight viable atypical lymphocytes, which are concerning for an underlying lymphoproliferative disorder, but histopathology was negative for definitive signs to confirm the diagnosis. (b) Gastric biopsy with CD3 immunohistochemistry (IHC) staining, obtained from repeat EGD. The IHC highlights numerous T-cells present in the ulcerated tissue.

The patient underwent a diagnostic EGD with a biopsy, which revealed multiple ulcerations and erosions in the gastric body, antrum, and fundus (Figure 2). Biopsies of these lesions showed multiple viable atypical lymphocytes but were negative for definitive signs of lymphoproliferative disorder (Figure 3a). Subsequent bone marrow biopsy was also negative, and analysis of the patient’s skin lesions and flow cytometry showed CD8-positive T-cell lymphoma, favoring primary cutaneous CD8+ aggressive epidermotropic cytotoxic T-cell lymphoma. Immunohistochemical stains of these cells further indicated that the patient’s pathology was primarily T-cell in origin as opposed to B-cell; stains for T-cell markers (CD3, 7, GATA3) were positive; and stains for B-cell markers (CD25, 30, ALK1) were negative; and flow cytometry was found to be within normal limits. Ki-67 revealed an overall 50% proliferation index indicating a moderately aggressive cancer with a poor prognosis, however, the patient’s lactate dehydrogenase (LDH) level was low. The patient was also assessed for possible viral causes of his diagnosis (human immunodeficiency virus (HIV), Epstein-Barr virus (EBV)), which were all negative.

**Figure 4:**
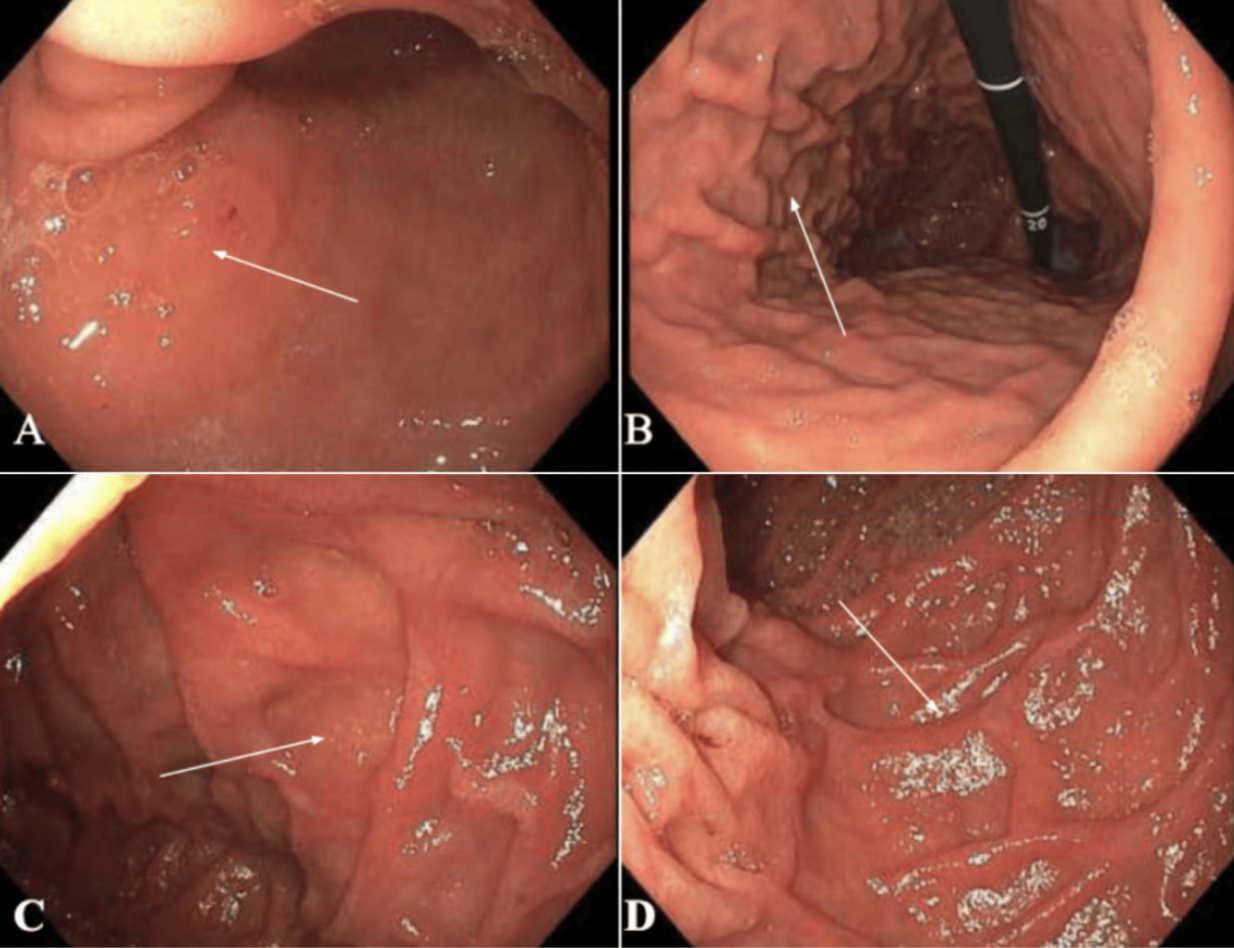
Repeat EGD with biopsy (a-b) Mild-moderate gastritis, characterized by congestion and erythema denoted by white arrows, in the gastric body with ulcers largely resolved from prior and otherwise showing healthy light pink gastric mucosa, (c-d) Mild duodenitis represented by erythematous mucosa, again denoted by white arrows, without active bleeding or stigmata of bleeding found in the second portion of the duodenum. Multiple biopsies were taken with cold forceps for histology and flow cytometry from the stomach and second portion of the duodenum. No evidence of a lymphoproliferative disorder was found on flow cytometry or histopathology and *Helicobacter pylori *testing was negative. EGD: esophagogastroduodenoscopy

Repeat EGD for further biopsy was performed due to the concern that the patient’s multiple ulcerations could be an extracutaneous manifestation of his pcAECTCL (given the marked uptake in the gastric region on PET scan despite the negative initial biopsy), as well as for further evaluation of the healing process. Repeat EGD showed gastritis with healing ulcers and duodenitis (Figure 4). Multiple biopsies were taken for pathology and flow cytometry. The histopathology of gastric biopsies was negative for *Helicobacter pylori* but showed T lymphocytes in the ulcerated tissue (Figure 3b). However, a definitive diagnosis of a lymphoproliferative disorder could not be made and the histopathology findings were found to be reflective of a focal mild chronic superficial gastritis. Flow cytometry of the biopsies was negative for a lymphoproliferative disorder, further supporting a non-malignant cause of the EGD findings. Subsequently, the patient underwent chemotherapy (CHOEP - cyclophosphamide, doxorubicin, etoposide, vincristine, and prednisone) for pcAECTCL, and he was scheduled for an allogeneic stem cell transplant to be performed once a donor is identified. A repeat PET scan showed that the hypermetabolism within the right anterior chest wall stomach seen in the prior study had resolved after ~2 months of chemotherapy. Additionally, the patient’s gastrointestinal symptoms subsided with the use of antacids for two months.

## Discussion

As established, pcAECTCL is a rare and poorly understood cutaneous lymphoma. It accounts for less than 1% of all cases of CTCL [5]. Patients frequently present with skin patches, plaques, and/or tumors with or without ulceration [5-7]. This patient’s skin manifestations began as a small nodule that progressed to ulcerated patches. The skin lesions can spread to other organs, such as the lung, testicles, central nervous system, and oral mucosa, without involving the lymph nodes [6].

The rarity of pcAECTCL and its extracutaneous manifestations make ascertaining the true cause of our patient’s gastric ulcers technically challenging. Given that the primary manifestation of pcAECTCL pathology is ulcerating skin lesions, it would logically follow that gastric mucosal ulcerations would be an extraintestinal manifestation of the disease via metastasis. The patient’s skin biopsy did indeed confirm CD8-positive T-cell lymphoma that was CD30 negative, favoring a diagnosis of primary cutaneous CD8-positive aggressive epidermotropic cytotoxic T-cell lymphoma. However, although the pathology report had some features suspicious for potential lymphoproliferative disorder (such as increased T-cells seen on histopathology of the repeat gastric biopsies), his flow cytometry of these biopsies was negative. The pathology report of the gastric fundus and body ulcers seen on EGD biopsies ultimately suggests that the ulcers were either inflammatory, a reaction to medication, or, less likely, ischemic as opposed to malignant in origin. No background intestinal metaplasia, dysplasia, or neoplasia was noted, indicating that the malignancy was not likely to have spread to the gastric mucosa and manifested in the form of our patient’s ulcers. Despite the lack of neoplastic findings seen on pathology, the PET scan showed increased gastric uptake, indicating that the ulcers were potentially lymphomatous in origin. This is further supported by the diminished gastric uptake seen on PET imaging following chemotherapy. 

These phenomena prompt further assessment to provide increased context to the patients’ findings. First, it has been reported that the false-negative rate for biopsy during EGD is estimated to be around 10%, including in patients with lesions that were followed over time, and can be as high as 23.3% in patients with synchronous lesions [8]. Factors contributing to this rate include the number of biopsies taken, the location of the ulcer (gastric body associated with more false negatives), underlying inflammatory conditions, or a cutaneous lymphoma that may obscure underlying malignancy or appear benign [8]. Simultaneously, there is also significant evidence to suggest that PET/CT has high false-positive rates across a wide array of suspected pathology, including lung cancers and lymphomas; this is most commonly seen in the setting of inflammatory lesions often necessitating further workup with biopsy [9,10]. It is not uncommon to see intense uptake on PET/CT, particularly in the gastric region, due to inflammatory pathology such as gastroesophageal reflux disease (GERD), gastritis - drug-induced, infectious, or ischemic; systemic inflammatory conditions, or other benign phenomena [11]. Finally, it has been observed in other cutaneous lymphoma variants, such as B-cell lymphomas, that these pathologies create a systemic inflammatory response/milieu that manifests as gastric ulcers and reflux in 10 and 50% of patients, respectively [12]. This is postulated to be due to the presence of cytokines and chemokines creating a pro-inflammatory environment leading to tissue damage and ulceration, even if the malignancy has not directly spread/metastasized to the tissue. Taken together, the findings in our patient could be explained by multiple converging factors - a false-positive PET, a false-negative biopsy, a systemic inflammatory state, or other iatrogenic causes, including a response to medications.

## Conclusions

This case describes an unusual presentation of a rare disorder. The case details a patient with primary cutaneous aggressive epidermotropic cytotoxic T-cell lymphoma (pcAECTCL), and gastric fundus/body ulcerations without metaplasia, dysplasia, or neoplasia on pathology. Cases of spread to the gastrointestinal tract in pcAECTCL have not been reported. However, very few cases of pcAECTCL have been recorded in the literature. This underscores the usefulness of this case in further understanding the varied manifestations of pcAECTCL. PcAECTCL is a rare, aggressive, poorly understood disease, necessitating further study.
